# Phylogeny and ultrastructure of *Myxobolus rangeli* n. sp. (Myxozoa, Bivalvulida), a histozoic parasite in Siluriformes fish from the Amazon region

**DOI:** 10.1590/S1984-29612023034

**Published:** 2023-06-19

**Authors:** Marcelo Francisco da Silva, Diehgo Tuloza da Silva, Elane Giese, Adriano Penha Furtado, Patricia Matos, Aline Medeiros Lima, Igor Hamoy, Edilson Matos

**Affiliations:** 1 Universidade Estadual da Região Tocantina do Maranhão – UEMASUL, Imperatriz, MA, Brasil; 2 Universidade Federal do Pará – UFPA, Belém, PA, Brasil; 3 Universidade Federal Rural da Amazônia – UFRA, Belém, PA, Brasil

**Keywords:** Myxozoa, freshwater fish, Pimelodus ornatus, Marajó island, Brazil, Myxozoa, peixe de água doce, Pimelodus ornatus, Ilha de Marajó, Brasil

## Abstract

A new species of *Myxobolus* parasitizing the arterial bulb and cardiac musculature of the freshwater fish *Pimelodus ornatus* Kner, 1858, from the Arari river in the municipality of Cachoeira do Arari, island of Marajó, Pará, Brazil, was described. In the present study, the observed prevalence of myxozoan parasites in the heart tissue of the hosts was 20% (6/30). The myxozoans observed had mature biconvex spores, slightly rounded, an anterior end with two pyriform polar capsules and a posterior end with very evident sporoplasm, measuring 8 ± 0.2 μmin length. The spore width was 5.8 ± 0.4 μm, with a thickness of 3.4 ± 0.2μm. The length of the polar capsules was 3.6 ± 0.3 μm and the width was 1.2 ± 0.2μm, with 6 to 7 turns of the polar filament. The divergences observed, regarding the morphometric and genetic structure of SSU rDNA, in relation to other Myxobolidae already described in the literature, confirm the description of the new species *Myxobolus rangeli* n. sp.

## Resumo

Descrição de uma nova espécie de *Myxobolus* que parasita o bulbo arterial e a musculatura cardíaca do peixe de água doce *Pimelodus ornatus* Kner, 1858, do rio Arari, no município de Cachoeira do Arari, ilha de Marajó, Pará, Brasil. No presente estudo, a prevalência observada de parasitas mixozoários no tecido cardíaco dos hospedeiros foi de 20% (6/30). Os mixozoários observados apresentavam esporos maduros biconvexos, levemente arredondados, extremidade anterior com duas cápsulas polares piriformes e extremidade posterior com esporoplasma bem evidente, medindo 8 ± 0,2 μm de comprimento. A largura do esporo foi de 5,8 ± 0,4 μm, com espessura de 3,4 ± 0,2 μm. O comprimento das cápsulas polares foi de 3,6 ± 0,3 μm e a largura foi de 1,2 ± 0,2 μm, com 6 a 7 voltas do filamento polar. As divergências observadas, quanto à estrutura morfométrica e genética de SSU rDNA, em relação a outros Myxobolidae já descritos na literatura, confirmam a descrição da nova espécie *Myxobolus rangeli* n. sp.

## Introduction

Myxozoans make up a group of extremely diverse metazoan parasites that develop endoparasitic interactions with aquatic organisms, especially fish (Lom & Dyková, 2006). Despite all the accumulated knowledge about the diversity of this group (Eiras et al., 2014; Abdel-Ghaffar et al., 2017; Okamura et al., 2018), the interactions and responses of the hosts to these organisms have only been partially revealed (Okamura et al., 2015; Naldoni et al., 2020).

Representatives of the genus *Myxobolus* Bütschli, 1882, are among the most frequently reported myxozoans that establish infectious relationships in freshwater fish. They are considered to be an important group of pathogens that can cause environmental and economic damage (Eiras et al., 2014; Molnár & Eszterbauer, 2015; Sindeaux-Neto et al., 2021). In the Amazon region, recent studies have described occurrences of *Myxobolus* sp. and *Myxobolus marajoensis* in the intestinal muscle of Siluriformes fish on the island of Marajó, Pará, Brazil (Abrunhosa et al., 2016, 2017).

Also in the state of Pará, Capodifoglio et al. (2019, 2020a, b) described *Myxobolus matosi*, *Myxobolus longissimus*, *Myxobolus colossomatis*, *Myxobolus arapiuns* and *Myxobolus pirapitingae*, parasitizing Characiformes hosts in the Tapajós river basin; and Sindeaux-Neto et al. (2021) published the description of the species *Myxobolus freitasi*, a parasite of the central nervous system of Gymnotiformes fish in the Peixe-Boi river basin, in the eastern portion of the Brazilian Amazon region.

The genus *Pimelodus* La Cépède, 1803, is the most species-rich group of fish in the family Pimelodidae in the Neotropical region (Garavello & Shibatta, 2007). Theseare small feral catfish commonly known as “mandi”. Myxozoan infections of the genus *Myxobolus* in hosts of this genus of Siluriformes were described by Cellere et al. (2002), in a study carried out in the Paraná river basin, Brazil. The present study deals with redescription andit used aspects of the ultrastructure and molecular biology of new species of *Myxobolus* that were described by Matos et al. (2014).

## Materials and Methods

Thirty fish of the species *Pimelodus ornatus* Kner, 1858, from the Arari river, were examined. They were caught in an area adjacent to the municipality of Cachoeira do Arari (01° 00'S; 48° 57' W) on the island of Marajó, in the state of Pará (Brazil). They were bought alive from artisanal fishermen, transported and kept in aquariums for up to 72 hours at the Carlos Azevedo Research Laboratory at the Federal Rural University of the Amazon (UFRA). They were then euthanized by means of anesthesia using tricaine methanesulfonate (MS222 Sandoz) at a concentration of 50 mg/L, inaccordance with the procedures approved by the ethics committee for animal experimentation. Following this, necropsy was performed to search for myxosporean infections, through dissection.

During the necropsy, the organs were examined under a stereomicroscope and tissue cysts were collected and analyzed by means of optical microscopy. The presence of parasites was verified.

Tissue fragments were subjected to the transmission electron microscopy (TEM) technique. Specimens were prepared by fixing them in 5% glutaraldehyde in 0.2 M sodium cacodylate buffer (pH 7.2) for 12 h at 4 °C, followed by washing in the same buffer for 12 h at 4 °C and post-fixation in 2% osmium tetroxide buffered with 0.2 M sodium cacodylate for 3 h. The material was dehydrated in an ascending series of ethanol concentrations, followed by propylene oxide, before incorporation into Epon resin. Semi-thin sections were stained with methylene blue, and ultra-thin sections with double contrast with uranyl acetate and lead citrate. These were observed under a JEOL 100CXII transmission electron microscope, which was operated at 60 kV in the Laboratory of Structural and FunctionalBiology of the Institute of Biological Sciences, at UFPA (LBEF/ICB/UFPA).

For scanning electron microscopy (SEM), fragments of parasitized tissues and free spores were fixed in 5% glutaraldehyde in 0.2 M sodium cacodylate buffer (pH 7.2) for 12 h at 4 °C, followed by washing in the same buffer for 12 h at 4 °C and post-fixation in 2% OsO_4_ buffered with 0.2 M sodium cacodylate for 3 h. The material was then dehydrated in an ascending series of ethanol concentrations, freeze-dried to a critical point, coated with gold and examined under aTESCANVega 3 LMU tabletop electron microscope at the Ultrastructure Laboratory of the UFRA Institute of Animal Health and Production (LU/ISPA/UFRA).

For molecular and phylogenetic analyses, myxosporid cysts were removed and fixed in 80% ethanol. DNA was extracted using the PureLink® Genomic DNA mini-kit (Invitrogen, USA), following the protocol provided by the manufacturer. The DNA samples were quantified in a Biodrop Duo spectrophotometer (Biodrop) and subjected to the polymerase chain reaction (PCR) technique in order to obtain the partial sequence of the small subunit ribosomal DNA (SSU rDNA), using primers that have been recommended in the literature. The ERIB1/ERIBI10 primer sets (Barta et al., 1997) were used in the first round of amplification, followed by the MC3/MC5 primers (Molnár et al., 2002), nested from the first round, and the ACT3f/ACT3r primers (Hallett & Diamant, 2001), semi-nested in the first pair with ERIB1 and ERIB10.

The amplifications were performed in a final reaction volume of 25 µl, containing 1 x ReddyMix PCR Master mixture (Thermo Scientific, USA), 75 mM Tris-HCl (pH 8.8), 20 mM KCl, 1.5 mM MgCl_2_, 0.2 mM of each nucleotide triphosphate (Thermo Scientific, USA), 10 pmol of each primer, 1.25 U of Taq DNA polymerase (Thermo Scientific, USA) and the DNA model (10-50 ng/µl). The reaction protocol for the ERIB1/ERIB10primers consisted of an initial extension at 95°C for 5 minutes, followed by 35 cycles of 95°C for 60 seconds, 56°C for 60 seconds and 72°C for 120 seconds, with a final extension step of 72°C for 10 min. For the other reactions, the reaction protocol was 95°C for 5 minutes, followed by 35 cycles of 95°C for 30 seconds, annealing temperatures of 56°C (nested PCR) or 58°C (semi-nested PCR) for 30 seconds and 72°C for 60 seconds, with a final extension step of 72°C for 10 min.

Subsequently, 3 µl of the PCR mixture was electrophoresed on 1% agarose gel with 1X Tris-borate-EDTA (TBE), stained with SYBR® Safe (Invitrogen, USA) and viewed under blue light. The PCR products were purified by means of DNA GFX ™ PCR and use of a gel strip purification kit (GE Healthcare, UK), in accordance with the manufacturer's instructions. The sequencing reactions were conducted using the Big Dye Terminator v3.1 cycle sequencing kit (Applied Biosystems, USA), following the manufacturer's instructions, in an ABI 3100 genetic analyzer (Applied Biosystems, USA).

The sequences obtained through this procedure were aligned in the BioEdit software (Hall, 2007) and any ambiguous bases were clarified using the respective chromatograms. Sequences of the SSU rDNA gene of myxozoan species that had previously been deposited in NCBI GenBank (Sayers et al., 2022) were aligned in Clustal X 1.8 (Thompson et al., 1997). Similarity scores greater than 80% in the Basic Local Alignment Search Tool (BLAST) were used as a criterion for selecting GenBank strings for inclusion in the analysis. The jModelTest software, version 0.1.1 (Guindon & Gascuel, 2003; Posada, 2008) was used to identify the best nucleotide replacement model for the data set.

Bayesian inferences were implemented in MrBayes, version 3.1.2 (Ronquist & Huelsenbeck, 2003).The Markov chain Monte Carlo method was used to search for two simultaneous executions of four chains of 10,000,000 generations, in which each 500^th^tree was sampled. A consensus tree was generated by means of the TreeAnnotator v1.8.4 tool, with a burn-in of 10%, and this was edited and plotted in FigTree v.1.4.3 (Rambaut et al., 2018). The reliability of the phylogenetic findings was verified by means of likelihood mapping analysis, in Tree-Puzzle 5.2 (Schmidt et al., 2002). Genetic distances were calculated in PAUP* 4.0b1 (Swofford, 2002), using the standard p parameter for the SSU rDNA gene.

Illustrative drawings were made from photomicrographs that had been obtained with the aid of a camera coupled to a computer.

## Results

**Figure 1:**
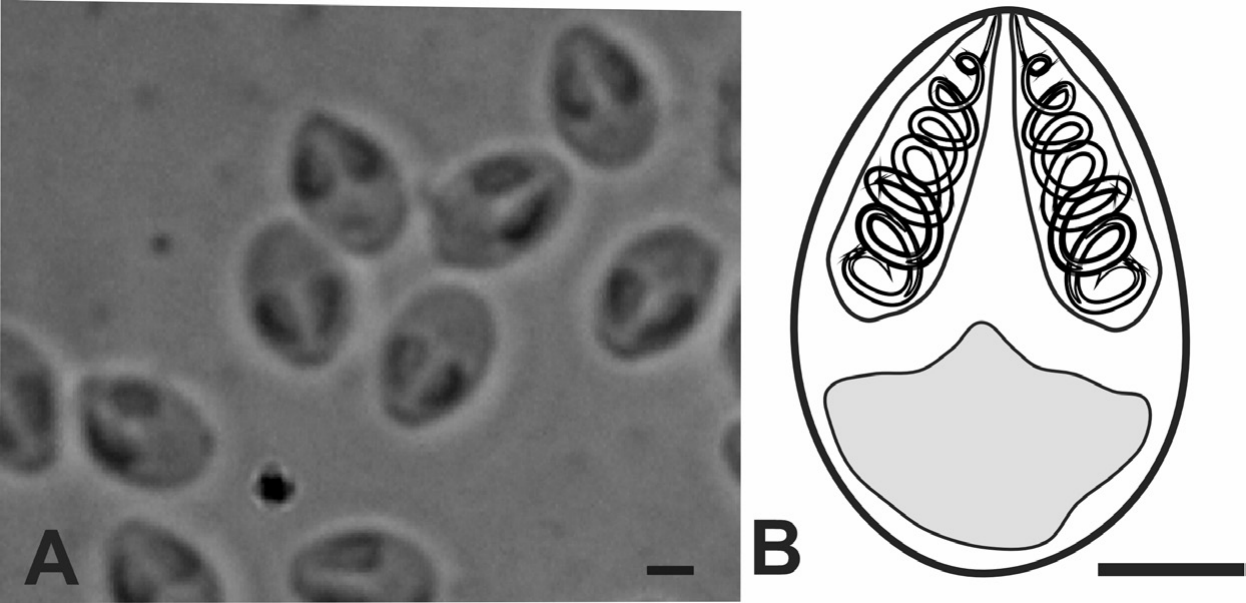
A: Photomicrography of the fresh spores. B: Diagram of spore of *Myxobolus rangeli* n. sp., in valvular view. Scale bar: 2 µm.

The macroscopic analysis showed the presence of whitish cysts, located in the cardiac musculature and in the region of the bulbus arteriosus of six specimens of *P.ornatus*, containing pyriform spores. The spore valves were symmetrical, with two equal polar capsules and a binucleated sporoblast (Figure 1).

**Figure 2:**
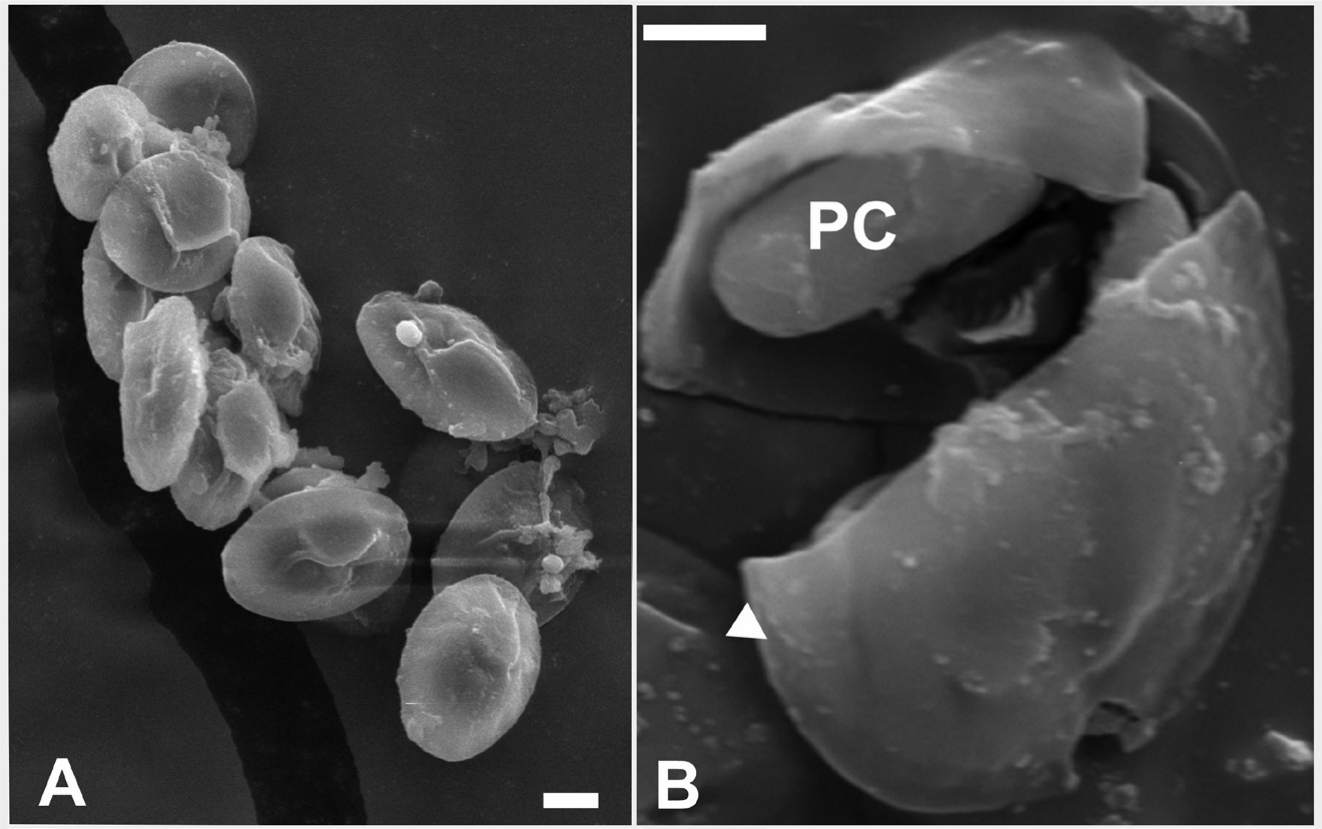
Scanning electron microscope (SEM) image of *M.rangeli* n. sp. A: View of myxospores after cyst rupture. B: View of the polar capsule (PC) and the valve wall (white arrow). Scale bar: 2 µm.

**Figure 3:**
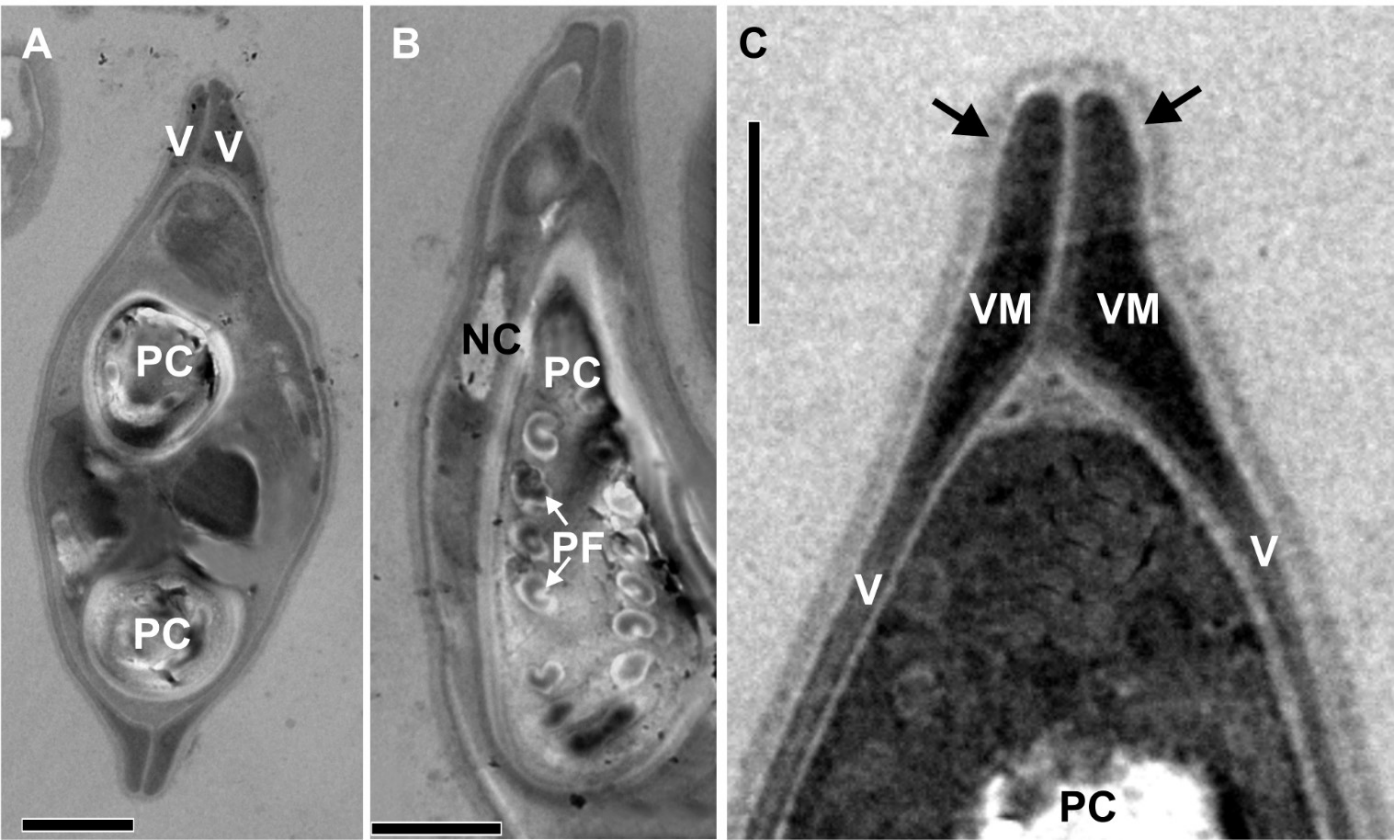
Electron micrographs of myxospores of *Myxobolus rangeli* n. sp., a parasite of the heart of *Pimelodus ornatus*. A: mature developmental stage of myxospores, with polar capsule (PC) and valves (V);scale bar: 2µm. B: longitudinal section of the myxospore, showing nucleus of capsulogenic cell (NC) and polar capsule (PC) with polar filaments (PF) (white arrow);scale bar: 2 µm. C: Detail of region of junction of the valves (v), showing valve-forming material (vm); PC − polar capsule;scale bar: 1µm.

Electron microscopy was performed onmature spores present in the bulbus arteriosus and in the heart muscle (Figure 2), and the presence of two identical conical valves was shown (Figures. 3A and 3C). The two polar capsules (PCs) were equal in size, pyriform and elongated, and they converged at the apex of the spore, with a polar filament (PF) presenting 6-7 turns (Figure 3B).

## Taxonomic Summary

Phylum: Cnidaria Verrill, 1865.

Unranked subphylum: Myxozoa Grassé, 1970.

Class: Myxosporea Bütschli, 1881.

Order: Bivalvulida Shulman, 1959.

Family: Myxobolidae Thélohan, 1892.

Genus: *Myxobolus* Bütschli, 1882

Species: *Myxobolus rangeli* n. sp.

Host type: *Pimelodus ornatus* Kner, 1858

Infection site: heart (bulbus arteriosus / cardiac muscle)

Description of cysts: The cysts were found to have an oval shape, showing a whitish color and averaging 286 µm (213-408) in length and 175 µm (122-245) in width.

Mature spores: The spores were 8.0 ± 0.2 µm long (n = 25), 5.8 ± 0.4 µm wide (n = 25) and 3.4 ± 0.2 µm thick (n = 15). The mature spores of *Myxobolus rangeli* n. sp. were relatively small compared with other representatives of the genus *Myxobolus*, and were described as parasitizing fish in Brazil. They were morphometrically closer to *Myxobolus flavus* Carriero et al. 2013 and *M. brycon* (Table 1).

**Table 1:** Morphometry of myxobolids of the genus *Myxobolus* parasites of fish from Brazil.

**Species**	**Spore Length**	**Spore width**	**Polar capsule length**	**Polar capsule width**	**Polar filament coils**	**References**
*Myxobolus rangeli* n. sp.	8 ± 0.2	5.8± 0.4	3.6 ± 0.3	1.2 ± 0.2	6–7	Present study
*M. aureus*	12.6 ± 0.5	8.3 ± 0.3	5.7 ± 0.3	2.9 ± 0.2	7–8	Carriero et al. (2013)
*M. brycon*	6.9 ± 0.6 (6.5–7.2)	4.2 ± 0.5 (3.9–4.8)	2.5 ± 0.7 (1.9–2.8)	1.9 ± 0.6 (1.7–2.5)	8–9	Azevedo et al. (2011)
*M. colossomatis*	11.8 (11.4–12.2)	6.9 (6.6–7.2)	6.0 (5.8–6.6)	6.0 (5.8–6.6)	7–8	Molnár & Békési (1993)
*M. cordeiroi*	11.1 ± 0.2	7.3 ± 0.1	5.4 ± 0.3	1.4 0.1	-	Adriano et al. (2009)
*M. cuneus*	10.0 ± 0.6	5.1 ± 0.3	5.7 ± 03	1.7 ± 0.2	8–9	Adriano et al. (2006)
*M. flavus*	9.2 ± 0.2	6.5 ± 0.3	4.5 ± 0.2	1.6±0.1	4–5	Carriero et al. (2013)
*M. heckelii*	12.7 (12.2–13.1)	6.6 (6.3–6.9)	2.9 (2.7–3.3)	1.7 (1.4–2.0)	4–5	Azevedo et al. (2009)
*M. maculatus*	21.0 (9.7–23.0)	8.9 (7.9–9.5)	12.7 (11.8–13.8)	3.2 (3.0–3.6)	14–15	Casal et al. (2002)
*M. metynnis*	12.9–13.5	7.5–8.3	5.0–5.5	5.0–5.5	8–9	Casal et al. (2006)
*M. myleus*	19.3 ± 0.5 (19–20)	8.3 ± 0.5 (7.5–9)	13.2 ± 0.4 (12.5–13.5)	3.0 ± 0.3 (2.5–3.5)	19–21	Azevedo et al. (2012)
*M. niger*	11.3 ± 0.4	6.8 ± 0.2	5.0 ± 0.3	2.0 ± 0.1	6–7	Mathews et al. (2016)
*M. piraputangae*	10.1 ± 0.5	8.7 ± 0.5	5.2 ± 0.4	3.0 ± 0.3	4–5	Carriero et al. (2013)
*M. umidus*	13.5 ± 0.7	7.8 ± 0.4	5.1 ± 0.4	2.7 ± 0.3	4–5	Carriero et al. (2013)

Locality type: Arari River, in the municipality of Cachoeira do Arari, Marajó Island, Pará, Brazil.

Prevalence: 6 out of 30 hosts examined (20%).

Type sample: Slides containing spores were obtained from the layer of the arterial bulb/cardiac muscle; these samples were deposited in the International Collection of Protozoan Samples of the National Institute of Amazonian Research (INPA), in Manaus, Amazonas, Brazil (catalog number: INPA 72).

Etymology: The species *Myxobolus rangeli* n. sp. is named in honor of Prof. Dr. Nello de Moura Rangel, who was an eminent professor and researcher in the fields of histology and embryology at the Federal University of Minas Gerais, Brazil (in memoriam).

Histopathology: The infection was characterized macroscopically by hypertrophy of the bulbus arteriosus and cardiac muscle that exhibited normal staining. Microscopic analysis of the bulbus arteriosus and cardiac muscle revealed infection by *Myxobolus rangeli* n sp., which was organized in the form of cysts on the pericardium, and with free spores in the myocardium. In association with areas of myocardial infection by free spores, there was degeneration and multifocal necrosis of muscle fibers, and discrete accumulation of mixed inflammatory infiltrate. It was concluded that the fish presented myocarditis secondary to infection by *Myxobolus rangeli* n sp.

Representative sequence: the SSU rDNA sequence of *M. rangeli* n. sp. has been deposited at GenBank under the accession number MT990755.

A partial sequence of 1,805 bp corresponding to the SSU rDNA was applied through sequencing spores of *Myxobolus rangeli* n. sp. found in the heart of *P. ornatus*.

**Figure 4:**
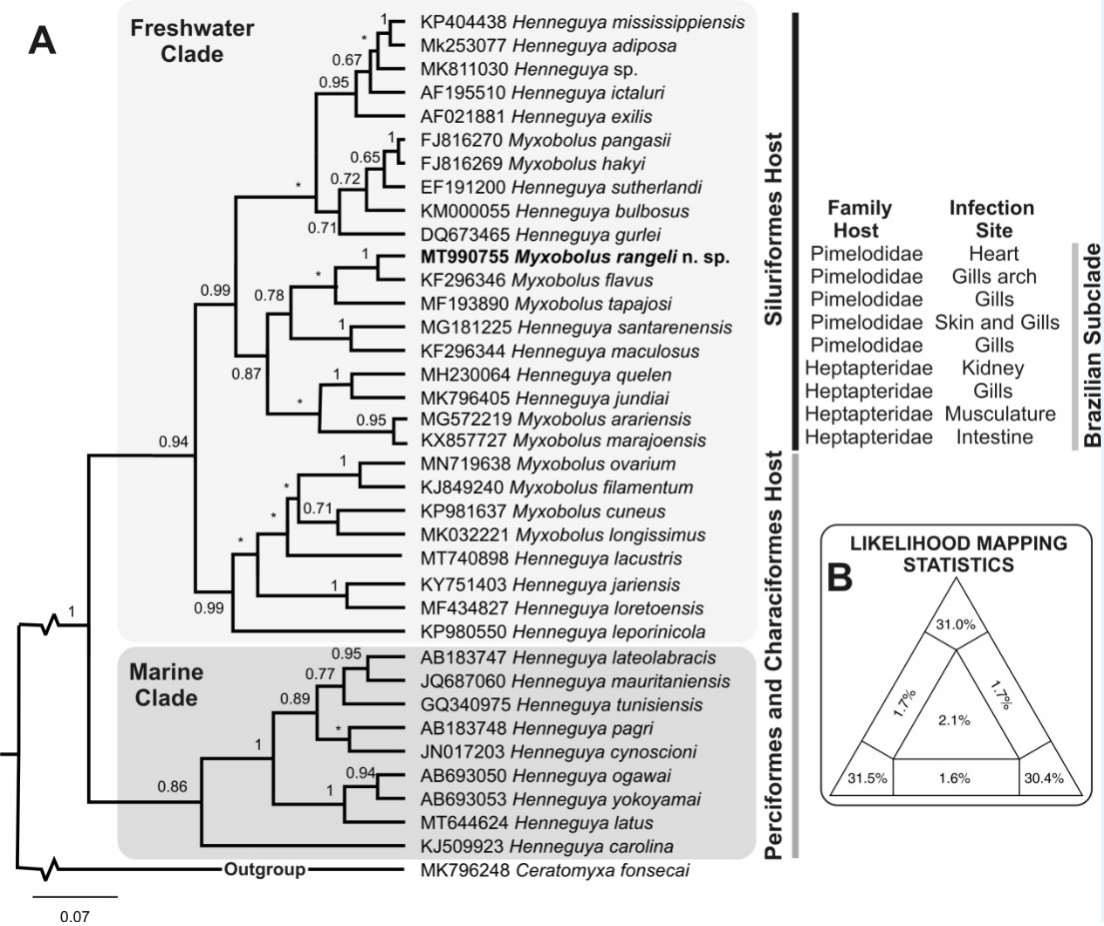
A: Results from Bayesian inference analysis on the partial sequence of the SSU rDNA gene of *Myxobolus rangeli* n. sp., combined with those of the 35 most relevant myxozoan species of the family Myxobolidae that have been registered in the NCBI GenBank, with a correspondence of over 80%;the numbers indicate Bayesian posterior probabilities. B: Likelihood mapping of the partial sequence of the SSU rDNA gene.

In the phylogenetic tree generated by means of Bayesian inferences, two main groups were formed (Figure 4), which both presented high nodal support (posterior probability). The clade formed by parasites of freshwater fish was divided into two subclades: the first comprised hosts of the orders Perciformes and Characiformes; while the second was formed by parasites of the genera *Henneguya* and *Myxobolus*, which are parasites of hosts of the order Siluriformes. *Myxobolus rangeli* n. sp. was present in the subclade of parasites of siluriform fish in South America, composed of a subclade of hosts of the family Pimelodidae, which presented high nodal support (posterior probability).

Among the parasites of this clade, *Myxobolusrangeli* n. sp. is phylogenetically close to *M.flavus* and *Myxobolus tapajosi* Zatti et al. 2018, which parasitize, respectively, *Pseudoplatystoma corruscans* Spix and Agassiz 1829 and *Brachyplatystoma rousseauxii* Castelnau, 1855, which are both Siluriformes living in the Amazon Basin, in Brazil. *Myxobolus flavus*, a branchial branch parasite, behaves like a sister species of *Myxobolus rangeli* n. sp.

Likelihood analysis, which generated 10,000 combinations of quartets, further confirmed the topology of the phylogenetic tree (Figure 4B). The alignment of the sequence contained 92.9% of the phylogeny within the tree. Only 5% of the quartets were partially resolved, while 2.1% were unresolved.

For pairwise comparisons, a new alignment was obtained, including only the species of *Myxobolus* that were parasites of Pimelodidae hosts, grouped with *M. rangeli* n. sp. The minimum genetic distance (p) was 4.2%, between *Myxobolus rangeli* n. sp. and *M. flavus* (KF296346), and the distance was 6.4% in relation to *M. tapajosi* (MF193890) (Table 2).

**Table 2:** Genetic distances (p-distances) between all Myxobolides species in the Pimelodidae fish subclate (Figure 3).

**Species**	**(1)**	**(2)**	**(3)**	**(4)**
**(1) *Myxobolus rangeli* n. sp. (MT990755)**				
(2) *Myxobolus flavus* (KF296346)	0.042			
(3) *Myxobolus tapajosi* (MF193890)	0.064	0.055		
(4) *Henneguya santarensis* (MG181225)	0.063	0.055	0.059	
(5) *Henneguya maculosus* (KF296344)	0.067	0.066	0.071	0.049

## Discussion

The morphology of the myxospores observed in the cardiac tissue of *Pimelodus ornatus* is consistent with the general characteristics defined for the genus *Myxobolus* Bütschli, 1882 (family Myxobolidae) (Lom & Dyková, 2006). *Myxobolus* infections in the cardiac musculature of different species of fish are rarely recorded in the scientific literature. Examples are the occurrence of *M. bulbocordis* in the heart of *Barbus sharpeyi* in Iran (Masoumian et al., 1996) and *M. muscularis* in the fibers of the heart and skeletal muscle of *Chelon ramada* in Portugal (Rocha et al., 2019).

Polyphyletism is a striking feature in most myxozoan genera (Kent et al., 2001; Azevedo et al., 2021). In addition to the combination of morphological and molecular analyses, factors related to the environment and tropism in relation to the host have been shown to be important in establishing the evolutionary relationships of this group (Fiala, 2006; Rocha et al., 2019). It was seenin the results described here that there were clear indications of grouping in relation to the type of environment, thus forming a well-defined clade of myxozoa that infect freshwater fish.

These results reinforce the hypothesis that host affinity is a strong evolutionary signal for myxobolids (Moreira et al., 2014; Vieira et al., 2018) and that the origin and irradiation of these parasites reflect the evolution of their hosts (Azevedo et al., 2021). The phylogeny presented here reinforces this finding, since the sequences analyzed were grouped according to the order and family of the hosts.

The morphological and morphometric characteristics of the spores and the partial sequence of the SSU rDNA gene obtained in the present study enabled description of *Myxobolus* parasites of the cardiac musculature of specimens of *Pimelodus ornatus*that were native to the island of Marajó, Brazil (Matos et al., 2014). The description of the new species, *Myxobolus rangeli* n. sp. contributes to knowledge of the biodiversity and phylogenetic relationships of myxozoan parasites of freshwater fish in the eastern portion of the Amazon region.

## References

[B001] Abdel-Ghaffar F, Abdel-Gaber R, Maher S, El Deeb N, Kamel R, Al Quraishy S (2017). Morphological and ultrastructural characteristics of *Myxobolus ridibundae* n. sp. (Myxosporea: Bivalvulida) infecting the testicular tissue of the marsh frog *Rana ridibunda* (Amphibia: Ranidae) in Egypt. Parasitol Res.

[B002] Abrunhosa J, Sindeaux-Neto JL, Santos AK, Hamoy I, Matos ER (2017). *Myxobolus marajoensis* sp. n. (Myxosporea: Myxobolidae), parasite of the freshwater catfish *Rhamdia quelen* from the Brazilian Amazon region. Rev Bras Parasitol Vet.

[B003] Abrunhosa JP, Silva MVO, Sindeaux-Neto JL, Santos PFS, Matos PS, Sanches OC (2016). Myxozoan infection in the muscle layer of the intestine of *Rhamdia quelen* from the Amazon River Basin, Brazil. Cienc Rural.

[B004] Adriano EA, Arana S, Alves AL, Silva MRM, Ceccarelli PS, Henrique-Silva F (2009). *Myxobolus cordeiroi* n. sp., a parasite of *Zungaro jahu* (Siluriformes: Pimelodidae) from Brazilian Pantanal: morphology, phylogeny and histopathology. Vet Parasitol.

[B005] Adriano EA, Arana S, Cordeiro NS (2006). *Myxobolus cuneus* n. sp. (Myxosporea) infecting the connective tissue of *Piaractus mesopotamicus* (Pisces: Characidae) in Brazil: histopathology and ultrastructure. Parasite.

[B006] Azevedo C, Casal G, Marques D, Silva E, Matos E (2011). Ultrastructure of *Myxobolus brycon* n. sp. (Phylum Myxozoa), parasite of the piraputanga fish *Brycon hilarii* (Teleostei) from Pantanal (Brazil). J Eukaryot Microbiol.

[B007] Azevedo C, Casal G, Matos P, Ferreira I, Matos E (2009). Light and electron microscopy of the spore of *Myxobolus heckelii* n. sp (Myxozoa), parasite from the Brazilian fish *Centromochlus heckelii* (Teleostei, Auchenipteridae). J Eukaryot Microbiol.

[B008] Azevedo C, São Clemente SC, Casal G, Matos P, Alves Â, Al-Quraishy S (2012). *Myxobolus myleus* n. sp. infecting the bile of the Amazonian freshwater fish *Myleus rubripinnis* (Teleostei: Serrasalmidae): morphology and pathology. Syst Parasitol.

[B009] Azevedo RK, Negrelli DC, Oliveira CP, Abdallah VD, Camara JPS, Matos ER (2021). Morphological and molecular analysis of *Henneguya lagunensis* n. sp. (Cnidaria, Myxosporea) parasitizing the gills of *Eugerres brasilianus* from Brazil. Parasitol Int.

[B010] Barta JR, Martin DS, Liberator PA, Dashkevicz M, Anderson JW, Feighner SD (1997). Phylogenetic relationships among eight *Eimeria* species infecting domestic fowl inferred using complete small subunit ribosomal DNA sequences. J Parasitol.

[B011] Capodifoglio KRH, Adriano EA, Naldoni J, Meira CM, Silva MRM, Maia AAM (2020). Novel myxosporean species parasitizing an economically important fish from the Amazon basin. Parasitol Res.

[B012] Capodifoglio KRH, Adriano EA, Silva MRM, Maia AAM (2019). The resolution of the taxonomic dilemma of *Myxobolus colossomatis* and description of two novel myxosporeans species of *Colossoma macropomum* from Amazon basin. Acta Trop.

[B013] Capodifoglio KRH, Meira CM, Silva MRM, Corrêa LL, Adriano EA, Maia AAM (2020). Morphology and molecular data of two novel cnidarian myxosporean (Myxobolidae) infecting *Piaractus brachypomus* from the Amazon basin. Acta Trop.

[B014] Carriero MM, Adriano EA, Silva MRM, Ceccarelli PS, Maia AAM (2013). Molecular phylogeny of the *Myxobolus* and *Henneguya* genera with several new South American species. PLoS One.

[B015] Casal G, Matos E, Azevedo C (2006). A new myxozoan parasite from the Amazonian fish *Metynnis argenteus* (Teleostei, Characidae): light and electron microscope observations. J Parasitol.

[B016] Casal G, Matos E, Azevedo C (2002). Ultrastructural data on the spore of *Myxobolus maculatus* n. sp. (phylum Myxozoa), parasite from the Amazonian fish *Metynnis maculatus* (Teleostei). Dis Aquat Organ.

[B017] Cellere EF, Cordeiro NS, Adriano EA (2002). *Myxobolus absonus* sp. n. (Myxozoa: Myxosporea) parasitizing *Pimelodus maculatus* (Siluriformes: Pimelodidae), a South American freshwater fish. Mem Inst Oswaldo Cruz.

[B018] Eiras JC, Zhang JY, Molnár K (2014). Synopsis of the species of *Myxobolus* Bütschli, 1882 (Myxozoa: Myxosporea, Myxobolidae) described between 2005 and 2013. Syst Parasitol.

[B019] Fiala I (2006). The phylogeny of Myxosporea (Myxozoa) based on small subunit ribosomal RNA gene analysis. Int J Parasitol.

[B020] Garavello JC, Shibatta OA (2007). A new species of the genus *Pimelodus* La Cépède, 1803 from the rio Iguaçu basin and a reappraisal of *Pimelodus ortmanni* Haseman, 1911 from the rio Paraná system, Brazil (Ostariophysi: Siluriformes: Pimelodidae). Neotrop Ichthyol.

[B021] Guindon S, Gascuel OA (2003). Simple, fast, and accurate algorithm to estimate large phylogenies by Maximum Likelihood. Syst Biol.

[B022] Hall T (2007). BioEdit. Biological sequence alignment editor for Win95/98/NT/2K/XP.

[B023] Hallett SL, Diamant A (2001). Ultrastructure and small–subunit ribosomal DNA sequence of *Henneguya lesteri* n. sp. (Myxosporea), a parasite of sand whiting *Sillago analis* (Sillaginidae) from the coast of Queensland, Australia. Dis Aquat Organ.

[B024] Kent ML, Andree KB, Bartholomew JL, El-Matbouli M, Desser SS, Devlin RH (2001). Recent advances in our knowledge of the Myxozoa. J Eukaryot Microbiol.

[B025] Lom J, Dyková I (2006). Myxozoan genera: definition and notes on taxonomy, life-cycle terminology and pathogenic species. Folia Parasitol (Praha).

[B026] Masoumian M, Baska F, Malnár K (1996). Description of *Myxobolus bulbocordis* sp. nov. (Myxosporea: Myxobolidae) from the heart of *Barbus sharpeyi* (Günther) and histopathological changes produced by the parasite. J Fish Dis.

[B027] Mathews PD, Maia AAM, Adriano EA (2016). Morphological and ultrastructural aspects of *Myxobolus niger* n. sp. (Myxozoa) gill parasite of *Corydoras melini* (Siluriformes: Callichthyidae) from Brazilian Amazon. Acta Trop.

[B028] Matos E, Videira M, Velasco M, Sanches O, São Clemente SC, Matos P (2014). Infection of the heart of *Pimelodus ornatus* (Teleostei, Pimelodidae), by *Myxobolus* sp. (Myxozoa, Myxobolidae). Rev Bras Parasitol Vet.

[B029] Molnár K, Békési L (1993). Description of a new *Myxobolus* species, *M. colossomatis* n. sp. from the teleost *Colossoma macropomum* of the Amazon River basin. J Appl Ichthyology.

[B030] Molnár K, Eszterbauer E, Székely C, Dán Á, Harrach B (2002). Morphological and molecular biological studies on intramuscular *Myxobolus* spp. of cyprinid fish. J Fish Dis.

[B031] Molnár K, Eszterbauer E, Okamura B, Gruhl A, Bartholomew JL (2015). Myxozoan evolution, ecology and development..

[B032] Moreira GSA, Adriano EA, Silva MRM, Ceccarelli OS, Maia AAM (2014). The morphological and molecular characterization of *Henneguya rotunda* n. sp., a parasite of the gill arch and fins of *Salminus brasiliensis* from the Mogi Guaçu River, Brazil. Parasitol Res.

[B033] Naldoni J, Pereira JOL, Milanin T, Adriano EA, Silva MRM, Maia AAM (2020). Taxonomy, phylogeny and host-parasite interaction of two novel *Myxobolus* species infecting *Bryconorthotaenia* from the São Francisco River, Brazil. Parasitol Int.

[B034] Okamura B, Gruhl A, Bartholomew JL, Okamura B, Gruhl A, Bartholomew JL (2015). Myxozoan evolution, ecology and development..

[B035] Okamura B, Hartigan A, Naldoni J (2018). Extensive uncharted biodiversity: the parasite dimension. Integr Comp Biol.

[B036] Posada D (2008). jModelTest: phylogenetic model averaging. Mol Biol Evol.

[B037] Rambaut A, Drummond AJ, Xie D, Baele G, Suchard MA (2018). Posterior summarization in Bayesian phylogenetics using Tracer 1.7. Syst Biol.

[B038] Rocha S, Casal G, Alves Â, Antunes C, Rodrigues P, Azevedo C (2019). Myxozoan biodiversity in mullets (Teleostei, Mugilidae) unravels hyperdiversification of *Myxobolus* (Cnidaria, Myxosporea). Parasitol Res.

[B039] Ronquist F, Huelsenbeck JP (2003). MrBayes 3: bayesian phylogenetic inference under mixed models. Bioinformatics.

[B040] Sayers EW, Bolton EE, Brister JR, Canese K, Chan J, Comeau DC (2022). Database resources of the national center for biotechnology information. Nucleic Acids Res.

[B041] Schmidt HA, Strimmer K, Vingron M, von Haeseler A (2002). TREE-PUZZLE: maximum likelihood phylogenetic analysis using quartets and parallel computing. Bioinformatics.

[B042] Sindeaux-Neto JL, Velasco M, Silva DT, Matos P, Silva MF, Gonçalves EC (2021). *Myxobolus freitasi* n. sp. (Myxozoa: Bivalvulida), a parasite of the brain of the electric knifefish in the Brazilian Amazon region. Rev Bras Parasitol Vet.

[B043] Swofford DL (2002). PAUP*. Phylogenetic analysis using parsimony (*and other methods).

[B044] Thompson JD, Gibson TJ, Plewniak F, Jeanmougin F, Higgins DG (1997). The CLUSTAL_X windows interface: flexible strategies for multiple sequence alignment aided by quality analysis tools. Nucleic Acids Res.

[B045] Vieira DHMD, Tagliavini VP, Abdallah VD, Azevedo RK (2018). *Myxobolus imparfinis* n. sp. (Myxozoa: Myxosporea), a new gill parasite of *Imparfinis mirini* Haseman (Siluriformes: Heptapteridae) in Brazil. Syst Parasitol.

